# Oral hygiene, prevalence of gingivitis, and associated risk factors among pregnant women in Sarlahi District, Nepal

**DOI:** 10.1186/s12903-018-0681-5

**Published:** 2019-01-05

**Authors:** D. J. Erchick, B. Rai, N. K. Agrawal, S. K. Khatry, J. Katz, S. C. LeClerq, M. A. Reynolds, L. C. Mullany

**Affiliations:** 1 Department of International Health, Johns Hopkins Bloomberg School of Public Health, Baltimore, MD USA; 2 Nepal Nutrition Intervention Project, Sarlahi (NNIPS), Krishna Galli, Lalitpur, Kathmandu Nepal; 3 Department of Dentistry, Institute of Medicine, Tribhuhvan University, Kathmandu, Nepal; 4 Department of Advanced Oral Sciences and Therapeutics, University of Maryland School of Dentistry, Baltimore, MD USA

**Keywords:** Nepal, Gingivitis, Oral health, Oral health behaviors, Dental care seeking behavior, Pregnancy

## Abstract

**Background:**

The oral health status of pregnant women in low-resource communities such as Nepal has not been well characterized. This sub-population is also of specific interest given associations between poor oral health and adverse pregnancy outcomes previously documented in other settings. We explored relationships between gingivitis and risk factors among pregnant women in rural Nepal.

**Methods:**

The design was a community-based, cross-sectional study in a sub-area of Sarlahi District, Nepal. Pregnant women < 26 weeks gestation underwent clinical periodontal exams conducted by community-based oral health workers. Exams included a full mouth assessment measuring bleeding on probing (BOP), probing depth (PD) (six sites per tooth), and gingival recession, the distance from the cemento-enamel junction to the free gingival margin (two direct sites per tooth). Data on participant risk factors were collected through household surveys, including demographic characteristics, oral health behaviors, care seeking, and health attitudes. Multivariable logistic regression modeling was used to assess relationships between gingivitis and risk factors.

**Results:**

We enrolled 1452 participants, of which 40% (*n* = 582) had signs of clinical gingivitis and 60% (*n* = 870) clinical health**.** Average participant age was 23. Most participants (88%) had never received oral health care. Participants averaged 10% of sites with BOP with most (79%) having ≥1 site with BOP. Nine percent of participants had ≥1 site with PD ≥4 mm, although very few participants (0.7%) had sites with PD ≥5 mm. Few participants (13%) had any recession (≥1 mm). In the final adjusted model, odds of gingivitis increased by 3% for each year of age (aOR 1.03, 95% CI 1.00, 1.06) and were higher for women of short maternal stature (< 150 cm) (aOR 1.43, 95% CI: 1.14, 1.79) and among women reporting cost to be a barrier to seeking dental care (aOR 2.13, 95% CI: 1.09, 4.15).

**Conclusions:**

Gingivitis was common and associated with age, maternal stature, self-reported high cost of dental care, and other risk factors among pregnant women in rural Nepal.

**Trial registration:**

ClinicalTrials.gov Identifier: NCT01177111 (Nepal Oil Massage Study) and NCT02788786 (Pilot Trial).

**Electronic supplementary material:**

The online version of this article (10.1186/s12903-018-0681-5) contains supplementary material, which is available to authorized users.

## Background

Periodontal disease includes a group of inflammatory conditions, typically initiated by oral bacteria, progressing from reversible accumulation of plaque and inflammation of gingival tissue (gingivitis) to irreversible breakdown of the supportive tissues of the teeth and eventually tooth loss (periodontitis) [1]. Globally, gingivitis is common and severe periodontitis affects 10 to 15% of adult populations [2]. Risk factors for periodontal disease include older age, poor oral hygiene, tobacco use, alcohol consumption, stress, malnutrition, and diabetes mellitus and other chronic diseases, although these have been evaluated primarily in populations in high-income countries [3]. Associations have been observed between periodontal disease and distal factors such as income and education, urban/rural location, race, and ethnicity [4]. Periodontal disease has been implicated as a potential cause of systemic conditions, such as adverse pregnancy outcomes [5].

Although periodontal health is recognized as an important component of general well-being, oral health remains neglected in many low- and middle-income countries and among high-risk and underserved sub-populations in some high-income countries [6, 7]. In many low-income countries, oral health facilities, equipment, and personnel are in short supply, severely limiting access to care, especially preventative services [7]. Nepal, for example, has only 2 dentists per 100,000 population, one of the lowest ratios among South Asian countries [8]. Few studies have assessed the oral health and oral hygiene practices of adult Nepalis. A review of four surveys conducted in the 1980s concluded that Nepal ranked among the bottom 15% of countries in terms of periodontal conditions [9]. A more recent national survey (2004) showed some improvement; Community Periodontal Index of Treatment Needs (CPITN) scores among 15–16 year-olds were 26% periodontal health (score 0), 8% bleeding on probing (BOP) (score 1), 61% BOP and calculus (score 2), 5% probing depth (PD) 4–5 mm (score 3), and 0% PD ≥ 6 mm (score 4). Among adults 35–44 years, the corresponding scores were 7% periodontal health (score 0), 3% BOP (score 1), 27% BOP and calculus (score 2), 48% PD 4–5 mm (score 3), and 16% PD ≥ 6 mm (score 4) [10–12].

The oral health status of pregnant women in low-resource communities such as Nepal has not been well characterized. This sub-population is also of specific interest given previously documented associations between poor oral health and adverse pregnancy outcomes [5, 13]. In this study, we explored relationships between demographic characteristics, oral hygiene practices, care-seeking, knowledge, and gingivitis among pregnant women in the Terai region of Nepal.

## Methods

Participants in this study were enrolled in the Nepal Oral Health Cohort Study (NOHCS), a community-based, prospective cohort study of gingivitis and adverse pregnancy outcomes. NOHCS participants were identified and determined eligible using the infrastructure of a large community-based randomized trial, the Nepal Oil Massage Study (NOMS) (NCT01177111), which was actively enrolling a population-based sample of pregnant women in this study area.

### Oral health examination

Oral health examinations were performed by five auxiliary nurse-midwives, with no previous experience in dentistry or clinical research, who were trained as “community-based oral health workers” for this study by an experienced dentist (NKA) at the Department of Dentistry, Institute of Medicine, Tribhuvan University, Kathmandu, Nepal. Training for the oral health workers included identification of plaque and calculus, signs of gingivitis, and measurement of PD, BOP, and gingival recession, the distance from the cemento-enamel junction (CEJ) to the free gingival margin (CEJ-GM). Oral health field workers were also trained in clinical research methods and ethics for human subjects research. Training lasted 3–4 weeks and included classroom instruction and practice of periodontal techniques under the guidance of the dentist. We estimated the validity of PD measurements of the oral health workers relative to the dentist and found that percent agreement, weighted kappa scores, and intraclass correlation coefficients, with an allowance of PD ±1 mm, exceeded 99%, 0.7, and 0.9, respectively, indicating an acceptable level of agreement.

Periodontal measurements were made using a color Williams probe (Hu-Friedy, Chicago, IL, USA). PD was measured on six sites per tooth (disto-, mid-, and mesial- aspects of buccal and lingual surfaces) and the CEJ-GM distance on two sites per tooth (mid- buccal and lingual aspects), excluding third molars. After probing each quadrant, the presence or absence of BOP was recorded for buccal and lingual surfaces of each tooth. PD values were recorded in millimeters from 1 to 10, rounded to the next higher whole number. CEJ-GM distances were recorded similarly, with values of 0 to 10 mm. If the free gingiva was coronal to the CEJ, the CEJ-GM measurement was recorded as 0. Clinical attachment loss (CAL) was calculated by summing the PD and CEJ-GM distance; the CEJ-GM distance was assigned a value of 0 for distal and mesial sites, where this measure was not collected, and these sites were not considered in measures of CAL. All scores were recorded on paper forms by a trained assistant, and electronically entered by experienced data entry operators.

Clinical periodontal disease was defined according to a classification scheme developed by the American Academy of Periodontology and European Federation of Periodontology to update the 1999 classification of periodontal diseases and conditions [14]. A case of clinical health was defined as a participant with all sites PD ≤3 mm and BOP < 10% [15, 16]. A case of clinical gingivitis was defined as a participant with BOP ≥10%, stratified as localized gingivitis (BOP 10–30%) and generalized gingivitis (BOP ≥30%) [15, 16]. Among localized and generalized gingivitis categories, we further stratified participants by those with ≥1 site with PD ≥4 mm. Our descriptive and inferential analyses compared participants with clinical health vs. clinical gingivitis. Other periodontal characteristics, including prevalence of potential clinical signs of mild periodontitis, were also described [17–20].

### Oral hygiene questionnaire

Data on participant demographics, oral hygiene practice, care-seeking, knowledge, and other characteristics were collected in the home, through a series of questionnaires administered over the course of pregnancy. Questionnaires were developed jointly by the authors and senior research staff, who are experienced in the conduct of community-based public health research and resident in the study population. Pre-tests of the questionnaires were conducted among study research staff, although the instrument’s validity and reliability were not formally evaluated. Questionnaires were administered in Maithili or Nepali language, depending on the participant’s primary language, in the home of participants by female study workers who were locally resident in the study area.

### Statistical analysis

Descriptive bivariate analyses between participant characteristics and the outcome, gingivitis, were evaluated using t-tests and logistic regression for continuous and binary/categorical variables, respectively. We ran a series of logistic regression multivariable models, sequentially including risk factor variables from four broad domains: maternal characteristics, oral hygiene behaviors, oral health knowledge and attitudes, and socioeconomic characteristics. Covariates were selected for inclusion in these regression models by examining bivariate relationships with gingivitis status using a cutoff of *p* < 0.10. Adjusted odds ratios (aOR) and 95% confidence intervals (CI) were calculated. All statistical analyses were performed in STATA 14.2 (StataCorp, College Station, TX, USA).

### Ethical review

This study received ethical approval from the Institutional Review Board at Johns Hopkins Bloomberg School of Public Health (Baltimore, USA) and the Ethical Review Board of the Nepal Health Research Council (Kathmandu, Nepal).

## Results

Between January 11, 2016, and November 26, 2016, among 1458 eligible pregnant women approached, 6 refused participation, leaving a total of 1452 participants. Of these, 32 were lost to follow-up (30 could not be contacted and 2 could not be interviewed because they had moved outside the study area) after the periodontal examination. Therefore, these participants provided data on their periodontal health but not on oral health risk factors for this analysis. Average participant age was 23.1 (SD: 4.7) ranging from 15 to 41, and the mean gestational age of pregnant women at enrollment was 14 weeks, ranging from 6 to 25 weeks (Table 1). About two-thirds (*n* = 934, 64%) of women had a normal BMI, 29% (*n* = 428) were underweight, and only 6% (*n* = 90) overweight or obese. Roughly three-quarters of participants (*n* = 1049, 72%) had at least one previous pregnancy, while 28% (*n* = 403) of women had not been pregnant before. Literacy among the study population was low (*n* = 671, 46%) and more than half of women had never attended school (*n* = 783, 54%). A majority of women lived in homes constructed of thatch, sticks, or bamboo (*n* = 897, 62%), versus wood planks, brick, or stone (*n* = 555, 38%). Women were likely to have some electricity available in the home (*n* = 1338, 92%). Nearly all women had access to a well for drinking water (*n* = 1446, > 99%). Almost half of women had no access to a latrine (*n* = 624, 43%).

**Table 1:** Demographic characteristics of participants in the oral hygiene study

Characteristic	All	Clinical health	Clinical gingivitis	Mean difference / OR (95% CI)^a^
Maternal age				
Year (mean, ± SD)	23.1 ± 4.7	22.8 ± 4.6	23.6 ± 4.8	***0.8 (0.3, 1.3)***
Maternal age (years)				
< 18	157 (10.8)	115 (13.2)	42 (7.2)	**0.51 (0.35, 0.74)**
18- < 35	1267 (87.3)	739 (84.9)	528 (90.7)	Ref
≥ 35	28 (1.9)	16 (1.8)	12 (2.1)	1.05 (0.49, 2.24)
Maternal height				
Cm (mean, ± SD)	150.8 ± 5.5	151.2 ± 5.4	150.2 ± 5.6	***−1.0 (−1.6, −0.4)***
Maternal weight				
Kg (mean, ± SD)	45.8 ± 7.1	46.1 ± 7.2	45.4 ± 6.8	− 0.7 (− 1.5, 0.0)
Maternal BMI				
Underweight	428 (29.5)	256 (29.4)	172 (29.6)	1.01 (0.80, 1.27)
Normal weight	934 (64.3)	560 (64.4)	374 (64.3)	Ref
Overweight or obese	90 (6.2)	54 (6.2)	36 (6.2)	1.00 (0.64, 1.55)
Ethnic group				
Hills (Pahadi)	106 (7.3)	54 (6.2)	52 (8.9)	Ref
Plains (Madeshi)	1346 (92.7)	816 (93.8)	530 (91.1)	0.67 (0.45, 1.00)
Gravidity				
First pregnancy	403 (27.8)	260 (29.9)	143 (24.6)	**0.78 (0.61, 0.99)**
1–3 previous pregnancies	872 (60.1)	511 (58.7)	361 (62.0)	Ref
≥ 4 previous pregnancies	177 (12.2)	99 (11.4)	78 (13.4)	1.12 (0.81, 1.54)
Maternal literacy				
No	781 (53.8)	455 (52.3)	326 (56.0)	Ref
Yes	671 (46.2)	415 (47.7)	256 (44.0)	0.86 (0.70, 1.06)
Maternal education (years)				
0	783 (54.0)	456 (52.5)	327 (56.2)	Ref
1–9	404 (27.8)	248 (28.5)	156 (26.8)	0.88 (0.69, 1.12)
≥ 10	264 (18.2)	165 (19.0)	99 (17.0)	0.84 (0.63, 1.11)
Electricity				
No	114 (7.9)	62 (7.1)	52 (8.9)	Ref
Yes	1338 (92.1)	808 (92.9)	530 (91.1)	0.78 (0.53, 1.15)
House construction material				
None, thatch, sticks, or bamboo	897 (61.8)	526 (60.5)	371 (63.7)	Ref
Wood planks, bricks, or stone	555 (38.2)	344 (39.5)	211 (36.3)	0.87 (0.70, 1.08)
House roof material				
None, plastic, thatch, or grass	111 (7.6)	58 (6.7)	53 (9.1)	Ref
Tile, tin, or concrete	1341 (92.4)	812 (93.3)	529 (90.9)	0.71 (0.48, 1.05)
Latrine				
No latrine	624 (43.0)	356 (40.9)	268 (46.0)	Ref
Brick, concrete, or pit latrine	828 (57.0)	514 (59.1)	314 (54.0)	0.81 (0.66, 1.00)

Data presented as No. (%) unless otherwise noted

^a^T-test or unadjusted odds ratio and 95% confidence interval as appropriate

Statistically significant for a mean difference are in bold italic

Statistically significant for an odds ratio are in bold

### Periodontal disease status

The majority of women (*n* = 1224, 84%) had all 28 teeth (ignoring third molars) (Table 2). Most participants (*n* = 1151, 79%) had at least one site with BOP, and participants averaged 10% of sites with BOP, the equivalent of 17 tooth sites (median = 9) per participant. Mean PD was 1.7 mm (SD: 0.3) and max PD ranged from 2 mm to 7 mm. Nine percent (*n* = 125) of participants had at least 1 site with PD ≥4 mm, although very few participants (*n* = 10, 0.7%) had sites with PD ≥5 mm. Mean CAL was equivalent to mean PD, and nearly all of CAL ≥4 mm was due to periodontal pocketing secondary to gingival enlargement, indicating very little recession of the gingiva in this population. Only 13% (*n* = 184) of participants had at least one site with recession.

**Table 2:** Clinical periodontal characteristics of participants in the oral hygiene study

Characteristic	No., %
Number of teeth^a^	
Mean number of teeth (mean ± SD)	27.7 ± 0.8
Clinical health and gingivitis	
Health (All sites PD ≤3 mm and BOP < 10%)	870 (59.9)
Gingivitis (BOP ≥10% and/or PD ≥4 mm)	582 (40.1)
- Localized gingivitis (BOP < 30%)	468 (80.4)
- Proportion with no sites PD ≥4 mm	373 (79.7)
- Proportion with ≥1 sites PD ≥4 mm	95 (20.3)
- Generalized gingivitis (BOP ≥30%)	114 (19.6)
- Proportion with no sites PD ≥4 mm	84 (73.7)
- Proportion with ≥1 sites PD ≥4 mm	30 (26.3)
Bleeding on probing (BOP)	
Percent of sites BOP (mean ± SD)	10.2 ± 12.2
No sites BOP	301 (20.7)
≥ 1 site BOP	1151 (79.3)
≥ 1 site BOP & BOP < 10%	621 (42.8)
BOP ≥10% & BOP < 30%	416 (28.7)
BOP ≥30%	114 (7.9)
Probing depth (PD)	
Mean PD (mm) (mean ± SD)	1.7 ± 0.3
Mean PD at direct sites (mm) (mean ± SD)	1.5 ± 0.2
Percent of sites PD ≥4 mm (mean ± SD)	0.2 ± 1.0
≥ 1 site PD ≥4 mm	125 (8.6)
≥ 3 sites PD ≥4 mm	40 (2.8)
≥ 1 site PD ≥5 mm	10 (0.7)
≥ 3 sites PD ≥5 mm	3 (0.2)
Clinical attachment loss (CAL)^b^	
Mean CAL at direct sites (mm) (mean ± SD)	1.7 ± 0.3
≥ 1 site recession ≥1 mm	184 (12.7)
≥ 1 site CAL ≥4 mm	206 (14.2)
≥ 3 site CAL ≥4 mm	72 (5.0)
≥ 1 site CAL ≥5 mm	65 (4.5)
≥ 3 site CAL ≥5 mm	14 (1.0)
≥ 1 site CAL ≥6 mm	21 (1.5)
≥ 3 site CAL ≥6 mm	3 (0.2)
Percent of CAL ≥4 mm due to pocketing (mean ± SD)	99.6 ± 1.5
Percent of CAL ≥4 mm due to recession (mean ± SD)	0.3 ± 1.5

^a^Excluding third molars

^b^The distance from the cemento-enamel junction to gingival margin was only measured for the direct site on the buccal and lingual surfaces

Sixty percent of participants (*n* = 870) fit the definition of clinical health and 40% (*n* = 582) clinical gingivitis. Among women with gingivitis, 80% (*n* = 468/582) had localized gingivitis and 20% (*n* = 114/582) generalized gingivitis. The proportion of sites with PD ≥4 mm in women with localized and generalized gingivitis were similar (*n* = 95/468, 20% and *n* = 30/114, 26%, respectively (*p* = 0.161)). Categorizing participants according to the CPITN, 20% (*n* = 295) of participants had periodontal health (score 0); 71% (*n* = 1032) BOP or BOP and calculus (scores 1 & 2)^1^; 8% (*n* = 123) PD 4–5 mm (score 3); and < 1% (n = 2) PD ≥6 mm (score 4) [21, 22].

### Oral pain status

Very few participants (7%, *n* = 107) reported having any oral pain (Table 3). Of those with pain, it was typically mild (*n* = 90/107, 84%), although 16% (*n* = 17/107) reported moderate or severe pain. Participants characterized their pain experience as toothache (*n* = 50/107, 47%), loose tooth (*n* = 18/107, 17%), sore or bleeding gums (n = 30/107, 28%), lesions in the mouth (*n* = 12/107, 11%), and jaw pain (*n* = 25/107, 23%) (multiple responses possible). Treatment sought for pain was typically increased teeth brushing or gargling with warm salt water. Of those that used medicine to treat pain, 80% (*n* = 16/20) of participants could not identify what type of medicine (multiple responses possible). Only 18% (*n* = 19/107) of participants with pain sought care from a health practitioner. One-quarter (*n* = 350, 25%) of participants reported having seen blood at some point in their life when cleaning their teeth, and almost half of those women (*n* = 161/345, 47%) saw blood within in the last week.

**Table 3:** Oral pain status of participants

Characteristic	All	Clinical health	Clinical gingivitis	OR (95% CI)^a^
Current oral pain status^d^				
None	1312 (92.5)	794 (93.2)	518 (91.4)	Ref
Mild	90 (6.3)	47 (5.5)	43 (7.6)	1.40 (0.91, 2.15)
Moderate or severe	17 (1.2)	11 (1.3)	6 (1.1)	0.84 (0.31, 2.27)
Type of pain^b^				
Toothache	50 (46.7)	29 (50.0)	21 (42.9)	0.75 (0.35, 1.61)
Loose tooth	18 (16.8)	7 (12.1)	11 (22.4)	2.11 (0.75, 5.95)
Sore or bleeding gums	30 (28.0)	10 (17.2)	20 (40.8)	**3.31 (1.36, 8.05)**
Lesions in the mouth	12 (11.2)	6 (10.3)	6 (12.2)	1.21 (0.36, 4.02)
Jaw pain	25 (23.4)	11 (19.0)	14 (28.6)	1.71 (0.69, 4.22)
Treatment sought for pain^b^				
Brushing	48 (44.9)	30 (51.7)	18 (36.7)	0.54 (0.25, 1.18)
Medicine	20 (18.7)	14 (24.1)	6 (12.2)	0.44 (0.15, 1.25)
Gargle with warm salt water	40 (37.4)	23 (39.7)	17 (34.7)	0.81 (0.37, 1.78)
Change eating or drinking habits	16 (15.0)	6 (10.3)	10 (20.4)	2.22 (0.74, 6.64)
Visited a health practitioner	19 (17.8)	13 (22.4)	6 (12.2)	0.48 (0.17, 1.39)
Other	20 (18.7)	9 (15.5)	11 (22.4)	1.58 (0.59, 4.19)
Saw blood when cleaning teeth (ever)^c^				
No	1069 (75.3)	713 (83.7)	356 (62.8)	Ref
Yes	350 (24.7)	139 (16.3)	211 (37.2)	**3.04 (2.37, 3.90)**
Saw blood when cleaning teeth (in last week)^c^				
No	183 (53.2)	86 (63.2)	97 (46.6)	Ref
Yes	161 (46.8)	50 (36.8)	111 (53.4)	**1.97 (1.26, 3.06)**
Clench or grind teeth while sleeping				
No	1352 (95.2)	813 (95.4)	539 (94.9)	Ref
Yes	68 (4.8)	39 (4.6)	29 (5.1)	1.12 (0.69, 1.84)
Wake up with tired jaws				
No	1327 (93.5)	798 (93.7)	529 (93.1)	Ref
Yes	93 (6.5)	54 (6.3)	39 (6.9)	1.09 (0.71, 1.67)

Data presented at No. (%) unless otherwise noted

^a^Unadjusted odds ratio and 95% confidence interval as appropriate

^b^Multiple responses possible

^c^Participants seeing blood in the last week are a subset of those reporting having ever seen blood while cleaning teeth

^d^Oral pain defined as none (no pain), mild (only a bother), and moderate or severe (prohibits daily activities)

Statistically significant for an odds ratio are in bold

### Risk factors

Age, height, and gravidity were associated with gingivitis at the *p* < 0.05 level. Most notably, gingivitis was more common in women who were older (OR 1.04, 95% CI: 1.02, 1.06 for each year of age) and women of short (< 150 cm) stature (clinical health 338/870 (38.9%), clinical gingivitis 274/582 (47.1%), OR 1.40, 95% CI: 1.13, 1.73). Gingivitis was less common in women who had no previous pregnancies (clinical health 260/870 (30.0%), clinical gingivitis 143/582 (24.6%), OR 0.78, 95% CI: 0.61, 0.99), although this association became insignificant when controlled for age (aOR 0.89, 95% CI: 0.68, 1.15). Ethnic group (Pahadi or Madeshi), house roof construction material, and presence of a latrine were associated with gingivitis at the *p* < 0.1 level. Several characteristics known from previous studies to be associated with gingivitis are not listed in Table 1 because the prevalence of these factors was reported at or near 0%, for example, smoking and other tobacco use, alcohol use, and hypertension.

### Dental hygiene behaviors

Nearly all participants reported teeth cleaning, most only once per day (*n* = 1030, 73%) and in the morning (*n* = 1407, 99%) (multiple responses possible) (Table 4). More than three quarters (*n* = 1113/1412, 79%), however, indicated that, optimally, a person should clean their teeth twice or more per day. Average teeth cleaning time was 10 (median = 5) minutes. Three-quarters (*n* = 1044, 74%) of participants used a toothbrush, and a large proportion reported using datiwan (दतिवन) (*n* = 626, 43%), a local teeth cleaning instrument fashioned from twigs of a variety trees (multiple responses possible). We identified at least 18 species of tree – including bamboo, neem, Indian rosewood, mulberry, and guava – that are used for datiwan in this community. Datiwan use was much more common among families originating in the plains region of Nepal (Madeshi ethnic group), where almost half of women (*n* = 618/1346, 46%) reported using datiwan, than the hills region (Pahadi group), where only 8% (*n* = 8/106) reported use (OR 10.39, 95% CI: 5.02, 21.55). More than half of participants (*n* = 817, 58%) used toothpaste, 16% (*n* = 229) toothpowder, and 28% (*n* = 393) neither toothpaste nor toothpowder (multiple responses possible). Of women that used toothpaste, 55% (*n* = 584) had a toothpaste brand that contained fluoride. Dental floss and oral rinse use were nearly non-existent (< 1%). Very few women reported changing any of their dental hygiene behaviors at the start of their current pregnancy. In general, these dental hygiene characteristics were not strongly associated with gingivitis; although, at the *p* < 0.1 significance level, the condition was more common in those who used datiwan (clinical health 359/870 (41.3%), clinical gingivitis 267/582 (45.9%), OR 1.21, 95% CI: 0.98, 1.49).

**Table 4:** Dental hygiene behaviors of participants

Characteristic	All	Clinical health	Clinical gingivitis	Mean difference / OR (95% CI)^a^
Cleaning frequency (times per day)				
1	1030 (72.6)	618 (72.6)	412 (72.7)	Ref
≥ 2	388 (27.4)	233 (27.4)	155 (27.3)	1.00 (0.79, 1.27)
Cleaning frequency (times per week)				
1–6	56 (3.9)	28 (3.3)	28 (4.9)	Ref
7–13	1148 (80.9)	702 (82.4)	446 (78.7)	0.64 (0.37, 1.09)
≥ 14	215 (15.2)	122 (14.3)	93 (16.4)	0.76 (0.42, 1.37)
Cleaning timing^b^				
Morning	1407 (99.2)	845 (99.4)	562 (98.9)	0.55 (0.17, 1.82)
Afternoon	172 (12.1)	96 (11.3)	76 (13.4)	1.22 (0.88, 1.68)
Evening	291 (20.5)	175 (20.5)	116 (20.4)	0.99 (0.76, 1.29)
After meals	239 (16.8)	140 (16.4)	99 (17.4)	1.07 (0.81, 1.42)
After sweets	16 (1.1)	9 (1.1)	7 (1.2)	1.17 (0.43, 3.16)
Cleaning duration				
Minutes (mean ± SD)	10.0 ± 10.3	10.1 ± 10.3	9.7 ± 10.3	−0.4 (−1.5, 0.7)
Cleaning duration (mins)				
1–2	90 (6.4)	50 (5.9)	40 (7.1)	Ref
3–10	1070 (75.7)	645 (76.2)	425 (75.0)	0.82 (0.53, 1.27)
11–29	105 (7.4)	65 (7.7)	40 (7.1)	0.77 (0.43, 1.36)
≥ 30	149 (10.5)	87 (10.3)	62 (10.9)	0.89 (0.53, 1.51)
Instrument use^b^				
Toothbrush	1044 (73.5)	635 (74.5)	409 (72.0)	0.88 (0.69, 1.12)
Datiwan	626 (43.1)	359 (41.3)	267 (45.9)	1.21 (0.98, 1.49)
Finger	210 (14.8)	126 (14.8)	84 (14.8)	1.00 (0.74, 1.35)
Brush replacement frequency (months)				
1–2	720 (69.8)	442 (71.1)	278 (68.0)	Ref
3–6	275 (26.7)	156 (25.1)	119 (29.1)	1.21 (0.91, 1.61)
≥ 7	36 (3.5)	24 (3.9)	12 (2.9)	0.79 (0.39, 1.62)
Dentifrice use (at least once per week)^b^				
Toothpaste	817 (57.5)	497 (58.3)	320 (56.3)	0.92 (0.74, 1.14)
Danta munjhan powder	229 (16.1)	138 (16.2)	91 (16.0)	0.99 (0.74, 1.32)
Charcoal	39 (2.7)	23 (2.7)	16 (2.8)	1.04 (0.55, 2.00)
Other	54 (3.8)	30 (3.5)	24 (4.2)	1.21 (0.70, 2.09)
Fluoride in toothpaste				
No	455 (42.8)	274 (42.2)	181 (43.6)	Ref
Yes	584 (54.9)	361 (55.6)	223 (53.7)	0.94 (0.73, 1.20)
Don’t know	25 (2.3)	14 (2.2)	11 (2.7)	1.19 (0.53, 2.68)
New behaviors adopted during pregnancy^b^				
More teeth cleaning	54 (3.8)	35 (4.1)	19 (3.3)	0.81 (0.46, 1.43)
Less teeth cleaning	5 (0.4)	4 (0.5)	1 (0.2)	0.37 (0.04, 3.35)
New instrument	28 (2.0)	13 (1.5)	15 (2.6)	1.75 (0.83, 3.71)
New dentifrice	8 (0.6)	6 (0.7)	2 (0.4)	0.50 (0.10, 2.48)

Data presented as No. (%) unless otherwise noted

^a^T-test or unadjusted odds ratio and 95% confidence interval as appropriate

^b^Multiple responses possible

### Dental health care seeking behaviors

While the overwhelming majority of participants (*n* = 1248, 88%) had never visited a dentist, those that had sought professional care (*n* = 171, 12%) most often indicated that doing so was prompted by toothache (*n* = 94/171, 55%) or dental caries (*n* = 106/171, 62%) (multiple responses possible) (Additional file 1: Table S1). Barriers to visiting a dentist included not knowing about dentists (*n* = 257, 18%) or where to seek care (*n* = 224, 16%), but a large proportion also indicated there was no need for them to visit a dentist (*n* = 1160, 82%) (multiple responses possible). Commonly reported sources of dental health information were family (*n* = 1103, 78%), radio (*n* = 760, 54%), school (*n* = 650, 46%), friends (*n* = 457, 32%), and television (*n* = 216, 15%). Likelihood of gingivitis was lower among women who reported receiving dental health information from radio (clinical health 474/852 (55.6%), clinical gingivitis 286/568 (50.4%), OR 0.81, 95% CI: 0.65, 1.00) or television (clinical health 145/852 (17.0%), clinical gingivitis 71/568 (12.5%), OR 0.70, 95% CI: 0.51, 0.95) and among women who said they had no need to visit the dentist (clinical health 709/852 (83.2%), clinical gingivitis 451/568 (79.4%), OR 0.78, 95% CI: 0.59, 1.02). Risk of gingivitis was significantly higher among women reporting that cost of dental was too expensive (clinical health 17/851 (2.0%), clinical gingivitis 24/568 (4.2%), OR 2.16, 95% CI: 1.15, 4.07).

### Dental health attitudes

Participants reported many different reasons why they cleaned their teeth (Additional file 1: Table S2). Most frequently mentioned barriers to more frequent cleaning included never being taught to clean their teeth (*n* = 518, 37%), not thinking additional teeth cleaning was necessary (*n* = 531, 37%), too much bother (*n* = 448, 32%), teeth are not dirty (*n* = 251, 18%), not enough time (*n* = 257, 18%), and no one else is doing so around me (*n* = 216, 15%) (multiple responses possible). Gingivitis was less common among women who said that they clean their teeth because they were taught to do so (clinical health 359/852 (42.1%), clinical gingivitis 210/568 (37.0%), OR 0.81, 95% CI: 0.65, 1.00). Gingivitis was also less common among those that reported that reasons preventing them from brushing their teeth included: cleaning their teeth does not help (clinical health 78/852 (9.2%), clinical gingivitis 36/567 (6.4%), OR 0.67, 95% CI: 0.45, 1.01) or is not necessary (clinical health 334/851 (39.3%), clinical gingivitis 197/568 (34.7%), OR 0.82, 95% CI: 0.66, 1.03). Gingivitis was more common among those that reported that reasons preventing them from brushing their teeth included: no one else around me cleans their teeth (clinical health 109/852 (12.8%), clinical gingivitis 107/567 (18.9%), OR 1.59, 95% CI: 1.19, 2.12) or cleaning teeth is too much of a bother (clinical health 252/852 (29.6%), clinical gingivitis 195/567 (34.4%), OR 1.25, 95% CI: 0.99, 1.57).

### Multivariable analysis

In the final regression model, the odds of gingivitis increased significantly by 3% (aOR 1.03, 95% CI: 1.00, 1.06) for each year of age (Table 5). Odds of gingivitis were lower among the Madeshi vs. Pahadi groups (aOR 0.57, 95% CI: 0.37, 0.88). Odds of gingivitis were 43% higher for women of short stature (< 150 cm) (aOR 1.43, 95% CI: 1.14, 1.79). Participant self-report that cost inhibits how often they visit the dentist was associated with increased risk of gingivitis (aOR 2.13, 95% CI: 1.09, 4.15). As reasons for why participants did not clean their teeth more often, higher odds of gingivitis were seen among those reporting no one else does (aOR 1.69, 95% CI: 1.24, 2.31) and lower odds among those reporting that teeth cleaning doesn’t help (aOR 0.53, 95% CI: 0.34, 0.83), and don’t think it’s necessary (aOR 0.78, 95% CI: 0.62, 0.99).

**Table 5:** Association between participant characteristics and clinical gingivitis status

	Clinical gingivitis OR (95% CI)
Model 1 – Maternal characteristics (*n* = 1452)	
Age	**1.03 (1.01, 1.06)**
Maternal stature (< 150 cm)	**1.42 (1.15, 1.75)**
Primiparous	0.89 (0.69, 1.17)
Model 2 – Model 1 + Oral hygiene behaviors (*n* = 1452)	
Age	**1.03 (1.01, 1.06)**
Maternal stature (< 150 cm)	**1.39 (1.12, 1.73)**
Primiparous	0.90 (0.69, 1.18)
Teeth cleaning frequency	0.71 (0.46, 1.10)
Use of datiwan to clean teeth	1.15 (0.92, 1.42)
Model 3 – Models 1–2 + Oral health knowledge & attitudes (*n* = 1417)	
Age	**1.03 (1.01, 1.06)**
Maternal stature (< 150 cm)	**1.43 (1.14, 1.78)**
Primiparous	0.93 (0.71, 1.23)
Teeth cleaning frequency	0.66 (0.38, 1.15)
Use of datiwan to clean teeth	1.10 (0.88, 1.38)
No need to visit dentist	0.82 (0.62, 1.09)
Too much money to visit dentist	**2.20 (1.13, 4.28)**
Radio a source of dental health information	0.93 (0.74, 1.18)
TV a source of dental health information	0.76 (0.55, 1.05)
Clean teeth because taught to do so	0.81 (0.65, 1.02)
Prevent cleaning - it’s a bother	**1.28 (1.01, 1.62)**
Prevent cleaning - no one else does	**1.70 (1.25, 2.32)**
Prevent teeth cleaning - doesn’t help	**0.53 (0.34, 0.82)**
Prevent teeth cleaning - don’t think it’s necessary	**0.79 (0.62, 1.00)**
Final model – Models 1–3 + Socioeconomic status (*n* = 1417)	
Age	**1.03 (1.00, 1.06)**
Maternal stature (< 150 cm)	**1.43 (1.14, 1.79)**
Primiparous	0.91 (0.69, 1.20)
Teeth cleaning frequency	0.70 (0.40, 1.21)
Use of datiwan to clean teeth	1.13 (0.90, 1.43)
No need to visit dentist	0.82 (0.61, 1.09)
Too much money to visit dentist	**2.13 (1.09, 4.15)**
Radio a source of dental health information	0.93 (0.74, 1.18)
TV a source of dental health information	0.74 (0.53, 1.03)
Clean teeth because taught to do so	0.83 (0.66, 1.05)
Prevent cleaning - it’s a bother	1.25 (0.99, 1.58)
Prevent cleaning - no one else does	**1.69 (1.24, 2.31)**
Prevent teeth cleaning - doesn’t help	**0.53 (0.34, 0.83)**
Prevent teeth cleaning - don’t think it’s necessary	**0.78 (0.62, 0.99)**
Madeshi cultural group	**0.57 (0.37, 0.88)**
Sturdy roof construction	0.85 (0.56, 1.31)
Latrine in home	0.91 (0.72, 1.15)

Statistically significant for an odds ratio are in bold

## Discussion

We found approximately 40% of women in this population to have gingivitis and 79% to have ≥1 site with BOP. Although bleeding on probing was highly prevalent, very few women had signs of periodontitis. Periodontal probing depths of > 3 mm were observed in 9% of women, consistent with documented increases in gingivitis and probing depths during pregnancy [23]. Participants had minimal recession, and CAL estimates, in both women with and without disease, were driven primarily by probing depth. This may be a result of the young age distribution of the population. CPITN scores from our study fit with the disease burden seen in the most recent national survey of periodontal disease in Nepal [10]. In other populations, higher levels of gingival inflammation and probing depths were observed among pregnant women during pregnancy [23–25].

Age was positively associated with periodontal disease, although many common risk factors for periodontal disease, such as smoking, alcohol use, and hypertension and other chronic diseases were low or nonexistent in this population. The ethnic composition of the study population was relatively homogenous, divided across the Pahadi group (people originally from the hills) and Madeshi group (people originally from the plains)) indigenous to Nepal. Risk factors for poor oral health that were prevalent in our population included rural location, low-socioeconomic status, and poor oral hygiene, specifically that women typically only cleaned their teeth once per day (sometimes with datiwan, rather than a toothbrush), few used fluorinated toothpaste, almost none flossed or used oral rinse, and access to professional dental health care was rare [26–28].

Some oral hygiene habits identified in this study were described similarly elsewhere, such as the practice of cleaning teeth only once per day (typically in the morning), but other habits were different, such as the high prevalence of datiwan use in our population, likely driven by behaviors common to the Madeshi ethnic group and the Terai region [29–31]. Datiwan use was the main driver of the high teeth cleaning time, as it is common practice in this population to use datiwan over an extended period during the morning routine. A large majority of women in this community had never visited a dentist or received any dental care [29]. A higher proportion of participants from this study reported having pain due to bleeding or swollen gums (relative to tooth pain) compared to those in an urban population in Eastern Nepal [30]. These findings are complimented by a qualitative study conducted in this population by our research team, which found that women were receptive to switching their routine to brushing twice daily with toothpaste when instructed by a health worker [32].

Although a wide range of potential risk factors for gingivitis were examined, other important factors, including some known to be causative, were not represented, including systemic conditions, such as diabetes mellitus or psychosocial factors, such as stress [33]. Although chronic diseases are likely under-diagnosed in this rural population, we suspect the prevalence of chronic disease to be low, given previous studies of risk factors in this population, and the young age of women in this study [34]. Undernutrition was not directly considered in the analysis, but was likely prevalent among women in the study population [35].

This study had several limitations. Attachment loss, a common measure of periodontitis, was not included in our outcome definition because it was not collected for all sites. Other indicators of periodontal disease, such as gingival and plaque scores, were not measured due to visit time constraints and variability in exam conditions, particularly in the amount of lighting. Although the validity of PD measurements for each community-based oral health worker was assessed relative to a dentist, reliability between or within the oral health workers was not evaluated.

## Conclusion

This study found 40% of women of childbearing age to have signs of gingivitis. Oral hygiene behaviors were less frequent than and varied from recommended practices and access to dental health services was uncommon. Age and short maternal stature were among the risk factors for gingivitis. These findings were consistent with the burden of periodontal disease measured by other studies in Nepal, although some important differences were identified in this population, including the prevalent use of datiwan for teeth cleaning [12, 31]. Data from this study will allow for evaluation of the role of periodontal disease in adverse pregnancy outcomes, such as preterm birth and low birth weight. Future studies could describe in further detail the severity and prevalence of periodontal disease, for example, by utilizing gingival and plaque indices, or evaluate the effectiveness of community-based interventions to prevent oral disease in women and mothers in Nepal.

## Additional file

Additional file 1: **Table S1.** Dental health care seeking behaviors of participants. **Table S2.** Dental health attitude of participants. (DOCX 26 kb)

## Abbreviations

aOR: Adjusted odds ratios
BMI: Body mass index
BOP: Bleeding on probing
CAL: Clinical attachment loss
CEJ: Cemento-enamel junction
CI: Confidence intervals
CPITN: Community Periodontal Index of Treatment Needs
GM: Gingival margin
NOHCS: Nepal Oral Health Cohort Study
NOMS: Nepal Oil Massage Study
OR: Odds ratio
PD: Probing depth
SD: Standard deviation

## Footnotes

1 Presence of calculus was not assessed by this study.
