# Supplementary material for: Oral hygiene, prevalence of gingivitis, and associated risk factors among pregnant women in Sarlahi District, Nepal
Source: BMC Oral Health. 2019 Jan 5;19:2. doi: 10.1186/s12903-018-0681-5 (PMC6321675; doi:10.1186/s12903-018-0681-5)
Supplement: Supplementary file 1 — Table S1. Dental health care seeking behaviors of participants. Table S2. Dental health attitude of participants. (DOCX 26 kb) [file 12903_2018_681_MOESM1_ESM.docx]

**Supplementary Table 1: Dental health care seeking behaviors of participants**

| **Characteristic** | | **All** | **Clinical health** | **Clinical gingivitis** | **OR (95% CI)**^*^ |
| --- | --- | --- | --- | --- | --- |
| **Dentist visits over lifetime** | |  |  |  |  |
|  | 0 | 1248 (87.9) | 746 (87.7) | 502 (88.4) | Ref |
|  | 1 | 75 (5.3) | 42 (4.9) | 33 (5.8) | 1.17 (0.73, 1.87) |
|  | ≥2 | 96 (6.8) | 63 (7.4) | 33 (5.8) | 0.78 (0.50, 1.20) |
| **Reason for last dentist visit**^~^ | |  |  |  |  |
|  | Regular check-up | 26 (15.2) | 18 (17.1) | 8 (12.1) | 0.67 (0.27, 1.63) |
|  | Tooth ache | 94 (55.0) | 58 (55.2) | 36 (54.5) | 0.97 (0.52, 1.81) |
|  | Gum soreness or bleeding | 20 (11.7) | 8 (7.6) | 12 (18.2) | 2.69 (1.04, 7.00) |
|  | Dental caries | 106 (62.0) | 69 (65.7) | 37 (56.1) | 0.67 (0.35, 1.25) |
|  | Other | 32 (18.7) | 19 (18.1) | 13 (19.7) | 1.11 (0.51, 2.43) |
| **Treatment at last dentist visit**^~^ | |  |  |  |  |
|  | Regular check-up | 75 (43.9) | 44 (41.9) | 31 (47.0) | 1.23 (0.66, 2.28) |
|  | Filling or crown | 31 (18.0) | 25 (23.8) | 6 (9.0) | 0.31 (0.12, 0.81) |
|  | Tooth extraction | 82 (47.7) | 49 (46.7) | 33 (49.3) | 1.11 (0.60, 2.05) |
|  | Prescribed medicine | 148 (86.0) | 90 (85.7) | 58 (86.6) | 1.07 (0.44, 2.62) |
|  | Prescribed oral rinse | 52 (30.2) | 29 (27.6) | 23 (34.3) | 1.37 (0.71, 2.65) |
| **Barriers to visiting the dentist**^~^ | |  |  |  |  |
|  | No need to visit a dentist | 1160 (81.7) | 709 (83.2) | 451 (79.4) | 0.78 (0.59, 1.02) |
|  | Don't know about dentists | 257 (18.1) | 149 (17.5) | 108 (19.0) | 1.11 (0.84, 1.46) |
|  | Don't know where to find a dentist | 224 (15.8) | 128 (15.0) | 96 (16.9) | 1.15 (0.86, 1.54) |
|  | Travel to dentist is too far or expensive | 49 (3.5) | 26 (3.1) | 23 (4.0) | 1.34 (0.76, 2.37) |
|  | Cost of dental care | 41 (2.9) | 17 (2.0) | 24 (4.2) | 2.16 (1.15, 4.07) |
|  | Not enough time to visit dentist | 42 (3.0) | 26 (3.1) | 16 (2.8) | 0.92 (0.49, 1.73) |
|  | A family member prevents dentist visit | 36 (2.5) | 18 (2.1) | 18 (3.2) | 1.52 (0.78, 2.94) |
|  | Nervous about visiting a dentist | 37 (2.6) | 23 (2.7) | 14 (2.5) | 0.91 (0.46, 1.79) |
| **Source of dental health information**^~^ | |  |  |  |  |
|  | Family | 1103 (77.7) | 669 (78.5) | 434 (76.4) | 0.89 (0.69, 1.14) |
|  | School | 650 (45.8) | 388 (45.5) | 262 (46.1) | 1.02 (0.83, 1.27) |
|  | Dentist or dental hygienist | 197 (13.9) | 118 (13.8) | 79 (13.9) | 1.00 (0.74, 1.37) |
|  | Other health care worker | 93 (6.5) | 53 (6.2) | 40 (7.0) | 1.14 (0.75, 1.75) |
|  | Friend | 457 (32.2) | 275 (32.3) | 182 (32.0) | 0.99 (0.79, 1.24) |
|  | Community meeting | 134 (9.4) | 86 (10.1) | 48 (8.5) | 0.82 (0.57, 1.19) |
|  | Radio | 760 (53.5) | 474 (55.6) | 286 (50.4) | 0.81 (0.65, 1.00) |
|  | TV | 216 (15.2) | 145 (17.0) | 71 (12.5) | 0.70 (0.51, 0.95) |
|  |  |  |  |  |  |
| Data presented as No. (%) unless otherwise noted | | |  |  |  |
| ^*^ Unadjusted odds ratio and 95% confidence interval | | |  |  |  |
| ^~^ Multiple responses possible | |  |  |  |  |

**Supplementary Table 2: Dental health attitude of participants**

| **Characteristic** | | **All** | **Clinical health** | **Clinical gingivitis** | **OR (95% CI)**^*^ |
| --- | --- | --- | --- | --- | --- |
| **Reasons for teeth cleaning**^~^ | |  |  |  |  |
|  | Make teeth feel clean | 1396 (98.2) | 839 (98.4) | 557 (98.1) | 0.84 (0.38, 1.87) |
|  | Make teeth look clean | 1386 (97.5) | 834 (97.8) | 552 (97.2) | 0.79 (0.40, 1.54) |
|  | Prevent caries | 1414 (99.5) | 848 (99.4) | 566 (99.6) | 1.67 (0.32, 8.63) |
|  | Prevent bleeding gums | 1408 (99.1) | 842 (98.7) | 566 (99.6) | 3.70 (0.82, 16.74) |
|  | Prevent ulcers | 1410 (99.2) | 845 (99.1) | 565 (99.5) | 1.78 (0.47, 6.75) |
|  | Prevent foul breath | 1408 (99.1) | 845 (99.1) | 563 (99.1) | 1.07 (0.35, 3.28) |
|  | I was taught I should | 569 (40.0) | 359 (42.1) | 210 (37.0) | 0.81 (0.65, 1.00) |
| **Things that prevent more teeth cleaning**^~^ | |  |  |  |  |
|  | Too much bother | 448 (31.5) | 253 (29.7) | 195 (34.4) | 1.24 (0.99, 1.56) |
|  | Not enough time | 257 (18.1) | 157 (18.4) | 100 (17.6) | 0.95 (0.72, 1.25) |
|  | Cost of toothbrush or paste | 73 (5.1) | 47 (5.5) | 26 (4.6) | 0.82 (0.50, 1.35) |
|  | No one else around me cleans their teeth | 216 (15.2) | 109 (12.8) | 107 (18.9) | **1.59 (1.19, 2.12)** |
|  | Teeth or gums hurt when I clean teeth | 64 (4.5) | 35 (4.1) | 29 (5.1) | 1.26 (0.76, 2.09) |
|  | Teeth are not dirty | 251 (17.7) | 159 (18.6) | 92 (16.2) | 0.85 (0.64, 1.12) |
|  | Cleaning teeth doesn't help | 114 (8.0) | 78 (9.1) | 36 (6.3) | 0.67 (0.45, 1.02) |
|  | I was never taught to clean teeth | 518 (36.5) | 312 (36.6) | 206 (36.3) | 0.98 (0.79, 1.23) |
|  | I don't think it necessary | 531 (37.4) | 334 (39.2) | 197 (34.7) | 0.82 (0.66, 1.03) |
|  | I forget to clean teeth | 148 (10.4) | 94 (11.0) | 54 (9.5) | 0.85 (0.60, 1.21) |
|  |  |  |  |  |  |
| Data presented as No. (%) unless otherwise noted. | | |  |  |  |
| ^*^ Unadjusted odds ratio and 95% confidence interval | | |  |  |  |
| ^~^ Multiple responses possible | |  |  |  |  |
